# Role of Neo-Sinus on Thrombogenicity of Aortic Valve Prostheses: Experimental Proof-of-Concept Study

**DOI:** 10.1007/s13239-025-00792-z

**Published:** 2025-07-14

**Authors:** Saskia Thoenissen, Ilona Mager, Claudio A. Luisi, Markus Mous, Thomas Schmitz-Rode, Ulrich Steinseifer, Johanna C. Clauser

**Affiliations:** 1 Department of Cardiovascular Engineering, Institute of Applied Medical Engineering, Medical Faculty, RWTH Aachen University Hospital, Forckenbeckstr. 55, 52074 Aachen, Germany; 2 Institute of Applied Medical Engineering, Medical Faculty, RWTH Aachen University, Aachen, Germany

**Keywords:** In-vitro testing, Neo-sinus, Transcatheter aortic valve replacement, Thrombogenicity, PIV measurements, Blood test

## Abstract

**Purpose:**

Transcatheter aortic valve replacement (TAVR) is the standard treatment for patients with aortic diseases at high surgical risk. Transcatheter heart valve prostheses (THV) are inserted into the aortic valve, creating a new area between the native and artificial leaflets. This area, known as neo-sinus, increases the thrombogenicity of THVs. But there is a lack of testing methods that evaluate thrombogenicity in vitro.

**Methods:**

To analyze the flow field within the native sinus and the neo-sinus, Particle Image Velocimetry (PIV) was performed with a thrombosis tester. Additionally, a comparative study was conducted with porcine blood on two polycarbonate urethane valves, with and without neo-sinus, respectively. Blood samples collected every hour were analyzed for platelet count, coagulation via ROTEM parameters, and plasma-free hemoglobin. Thrombus formation was detected optically.

**Results:**

The PIV measurements yield a physiological flow field in the aortic root that were consistent with those reported in literature. The analyzed blood parameters reveal no obvious difference between the valve with neo-sinus and the valve without. A higher amount of thrombus material for the valve with neo-sinus was found.

**Conclusion:**

The visualized flow field shows low velocities and stagnation zones due to the presence of native leaflets. Clot formation at the heart valve prostheses are in accordance with in-vivo findings. The benchmark of the two valves indicates an increased thrombogenic potential due to the neo-sinus. The thrombosis tester simulates the natural environment after TAVR. Thereby, newly developed THVs can be evaluated in vitro and consequently optimized regarding their thrombogenicity.

**Supplementary Information:**

The online version contains supplementary material available at 10.1007/s13239-025-00792-z.

## Introduction

Due to the constantly aging society, the number of cardiovascular diseases is constantly increasing. For patients suffering from severe symptomatic aortic stenosis, the replacement of the diseased aortic valve is inevitable. The standard treatment for patients at high risk for surgical aortic valve replacement (SAVR) is the transcatheter aortic valve replacement (TAVR), a minimally invasive procedure in which the artificial prosthesis is inserted into the native valve. This treatment has also developed as the standard therapy for patients over the age of 75 years with low surgical risk [1–4]. However, the risk for thromboembolic morbidity remains higher after TAVR compared to surgical biological prostheses. This morbidity may be due to the higher incidence of subclinical leaflet thrombosis found in the aortic root after TAVR, as proved in several high-resolution computed tomography (CT) studies [5–9]. For instance, Chakravarty et al. showed that the incidence of subclinical leaflet thrombosis is higher with transcatheter heart valve prosthesis (THV) (13%) compared to surgical bioprosthetic valves (4%) [5].

**Fig. 1:**
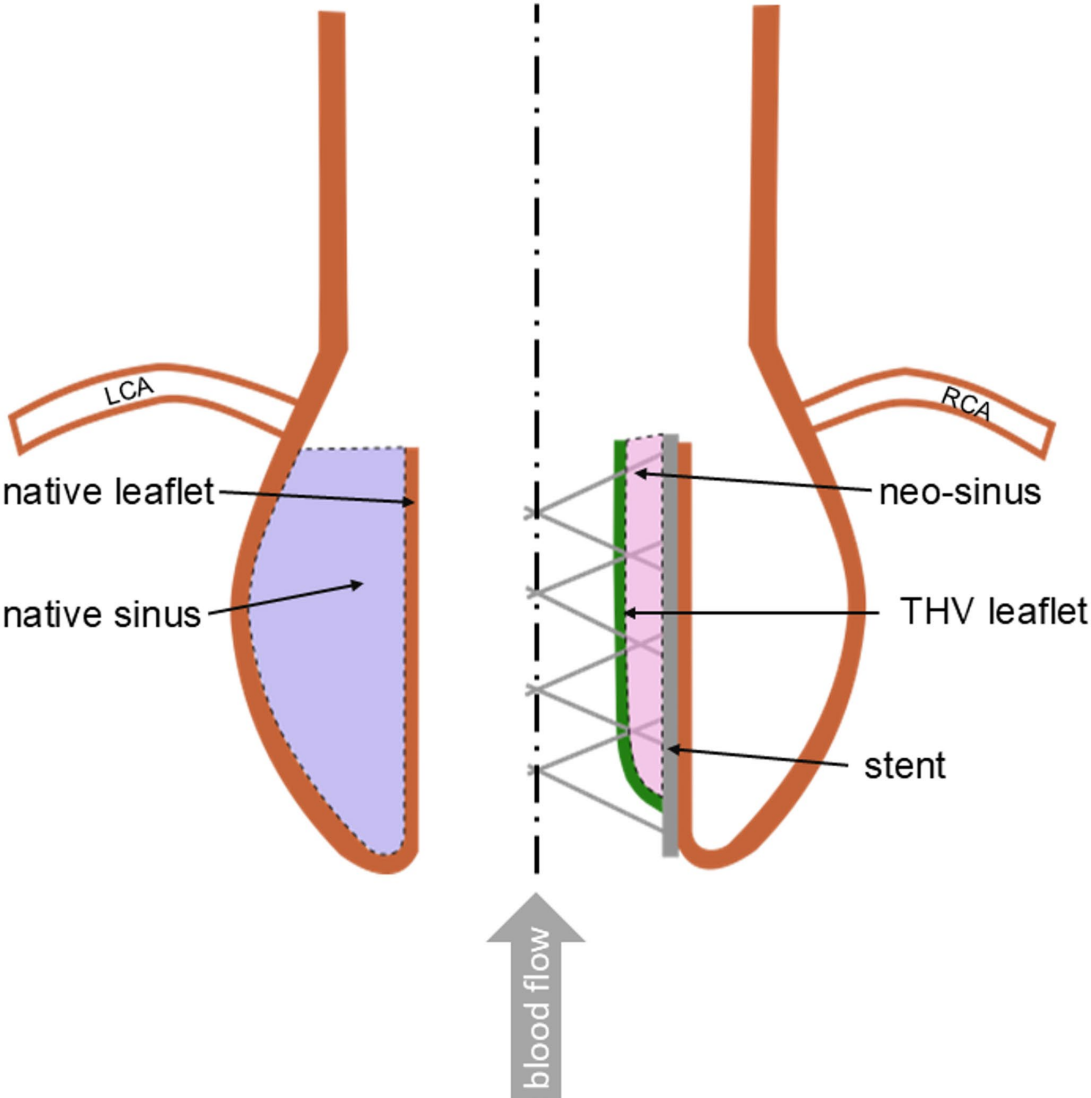
Schematic of the aortic root (LCA, left coronary artery; RCA, right coronary artery)

In TAVR, the THV is crimped in a catheter that is inserted into the vascular system towards the diseased aortic valve. There, the THV is expanded and pushes the native aortic leaflets against the aortic wall. As a result, a new area is created between the native leaflets and the artificial ones, called neo-sinus (Fig. 1).

The neo-sinus alters the flow dynamics in the aortic root leading to subclinical thrombus formation as demonstrated by several in-vitro studies using particle image velocimetry (PIV) and computational fluid dynamics (CFD) simulation [10–13]. In an in-vitro study by Vahidkhah et al., the authors measured the blood residence time (BRT) at the THV leaflet and compared it to surgical aortic valve (SAV). The authors’ CFD simulation showed that the BRT on THV leaflets was significantly longer than on surgical valves during all stages of the cycles [13]. Longer BRT and flow stasis in the aortic root are assumed to be the main factors for subclinical leaflet thrombosis. To minimize the thrombogenic risk for prosthetic heart valves (PHV), it has become standard to evaluate their thrombogenicity in an early phase of development using in-vitro test systems with blood or blood analog fluids, PIV or CFD measurements [13, 14]. However, few adequate testing systems exist so far that replicate the natural environment of an aortic valve after TAVR with blood as test fluid.

Only a few in-vitro test systems use blood as it is mandatory in the recent standard DIN EN ISO 5840-1:2021 [15]. One in-vitro test method that fits the standard for SAV was developed by Linde et al. [17]. They used mechanical aortic prostheses and polycarbonate urethane (PCU) valves to verify the testing system for SAV. Since the Thrombosis Tester of the Helmholtz Institute Aachen 3 (THIA 3) was developed for SAVs, the integration of aortic leaflets and therefore the neo-sinus in the test chamber was not considered. However, the replication of the neo-sinus and a validation of the flow field and clot formation is still missing for the THIA 3. Additionally, to evaluate the thrombogenicity of THVs with the THIA 3, the integration of the neo-sinus is essential and now mandatory in the revised standard DIN EN ISO 5840:3-2021.

In this study, the role of the neo-sinus on the thrombogenicity of aortic heart valves is evaluated in a proof-of-concept blood study. For this purpose, one blood test is conducted in the THIA 3, using two test chambers, one with neo-sinus and the other one without neo-sinus. To validate that the flow field corresponds to a physiological one in the case of the presence of native leaflets, PIV measurements visualize the flow dynamics in the native and neo-sinus. Thereby, higher thrombus formation in the presence of native leaflets and therefore the neo-sinus could be attributed to the altered flow dynamics in the aortic root.

## Materials and Methods

### Test Setup

**Fig. 2:**
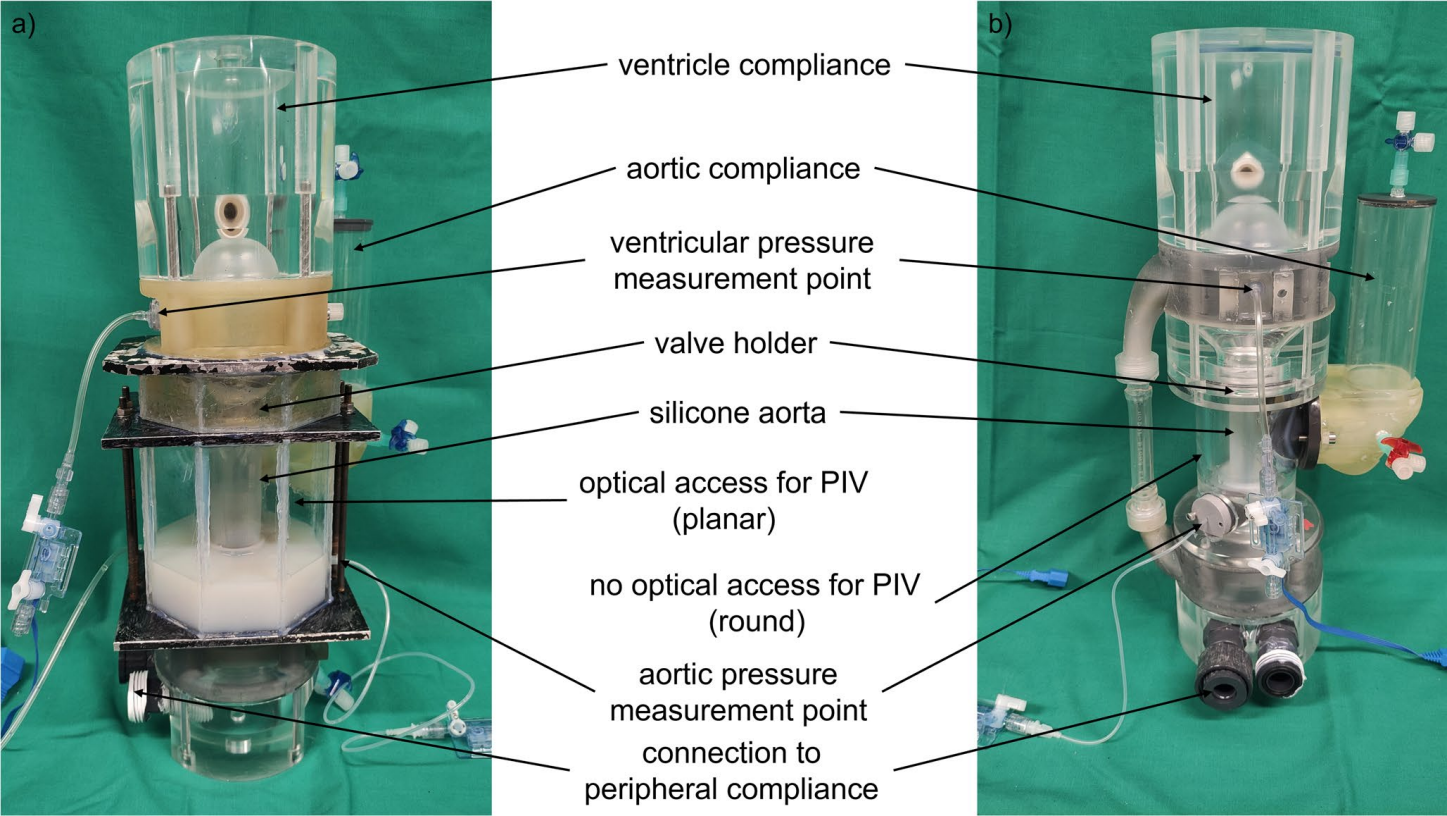
THIA 3 for (**a**) PIV measurements and (**b**) blood tests

The base for the test system is the Thrombosis Tester of the Helmholtz Institute Aachen 3 (THIA 3) that was developed for testing artificial aortic prostheses and was validated with mechanical and PCU prostheses before [17]. The test system allows to investigate the interaction between blood, the surface of the prosthesis and the blood flow through it. To analyze the flow dynamics and flow paths in the aortic root downstream the valve and to correlate it with formed thrombi, a comparable test bench as for the blood test was constructed for Particle Image Velocimetry (PIV) measurements (Fig. 2).

The design of both testing systems was adopted from Linde et al. and is described elsewhere [17]. Briefly, the test bench is composed of a driving unit and a test chamber. The test chamber consists of a ventricle, a prosthesis holder, a flexible aorta, and a water-filled ventricular, aortic, and peripheral compliance, respectively. The test fluid flows inside the ventricle and the aorta inlay, which is made from silicone (Elastosil RT 620 A/B, Wacker Chemie AG, Germany). For each test, new inlays are produced manually in a casting process. In contrast to the blood test bench, the chamber around the silicone aorta was designed as a prism with a regular octagon as base area to permit optical access for PIV.

**Fig. 3:**
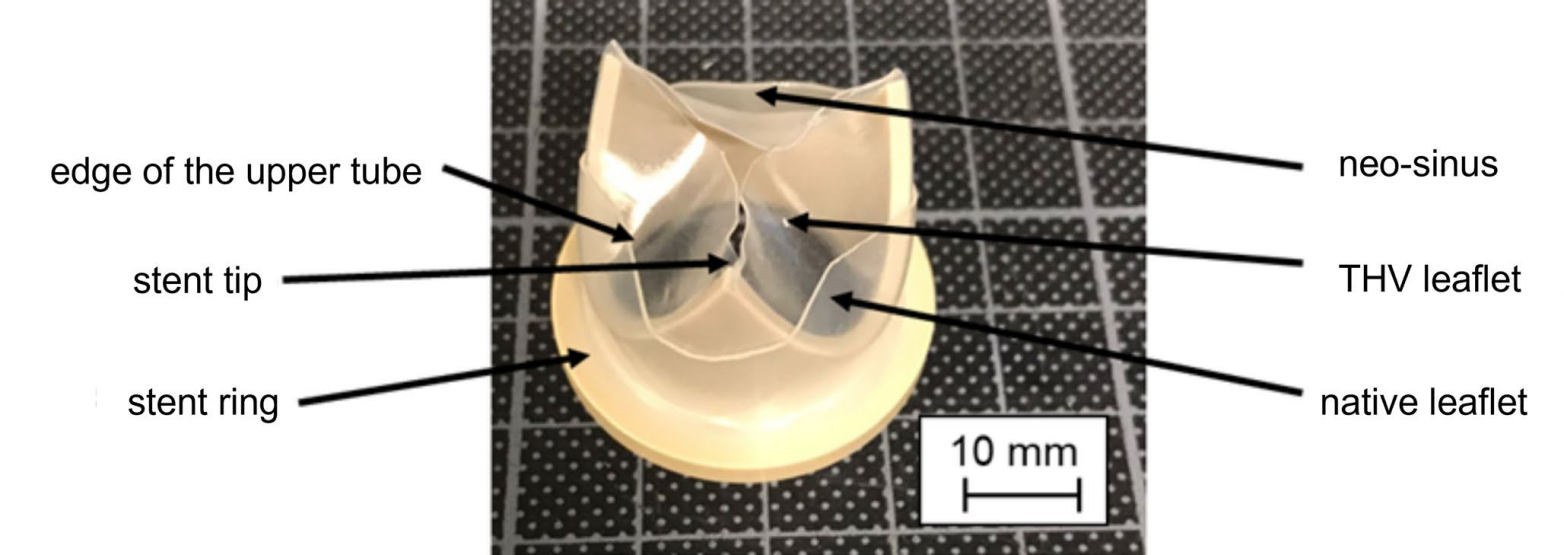
In-house designed PCU valve with native leaflets

As testing device, an in-house designed PCU valve was chosen due to available reference blood and PIV data [17, 18]. Native leaflets were reproduced so that the neo-sinus is formed. The leaflets were modeled by a polycarbonate urethane (PCU) tube, with its upper edge shaped like native commissures, and pulled over the PCU valve. This created the neo-sinus between the PCU tube and the leaflets of the prosthesis, both made of PCU (Fig. 3). The aortic root geometry is designed by Bronstein-Semendjajev epitrochoid and is described elsewhere. [18]

Blood and PIV experiments were conducted under physiological conditions with an aortic pressure of 120/80 mmHg and a cardiac output of 3.5 L/min as it was described in the protocol by Linde et al. [18] and is in line with the ISO 5840 requirements. The resulting stroke volume was 50 ml. The beat rate of 70 bpm and the systole/diastole ratio of 35% were simulated by the stroke and the velocity of the linear piston. The aortic and ventricular pressure as well as the pressure in the aortic and peripheral compliance were measured using pressure sensors (DPT6000, CODAN pvb Medical GmbH, Germany). In contrast to Linde et al., the pressure measurement of the aortic and ventricular pressure was done directly. The direct pressure measurement increases robustness and accuracy since it is less susceptible to errors due to assumptions. For the ventricular pressure, the filling port is used as pressure measurement point. The aortic pressure is measured using a perfusion line (B. Braun, Germany), which is pushed through the silicone aortic wall (see figure S1 in the supporting information). The flow through the bypass was recorded by ME9PXL16 ultrasonic flow probes (Transonic Europe BV, Netherlands). The data acquisition of the hydrodynamic parameters was performed with LabView (National Instruments, USA).

### PIV Measurements

To validate the maintenance of physiological flow conditions in the adapted test chamber with neo-sinus, a PIV measurement was performed with the PCU valve prototype and the implemented neo-sinus. The test chamber as well as the ventricle and aorta inlay were filled with a water-glycerol solution with 49.5 wt% glycerol to match the viscosity of blood with 3.6 mPa s and the refractive index of 1.4 at a temperature of 37 °C. Additionally, fluorescent polystyrene microparticles (diameter 10.5 μm; ILA GmbH, Germany) were added.

**Fig. 4:**
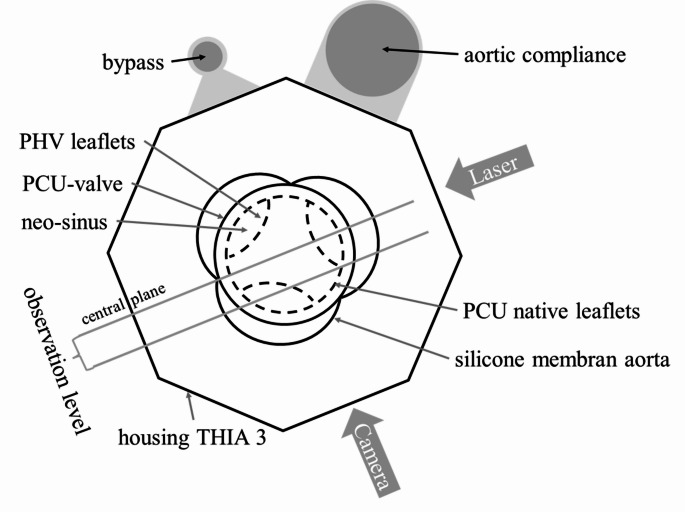
Arrangement of test device, test bench, camera and laser in the conducted PIV experiment

For PIV recordings, an EverGreen 70 mJ double pulse Nd: YAG laser (Quantel, France) operating at a wavelength of 532 nm was used. The central plane, which is orthogonal to the valve plane, was chosen for the flow through the valve in total. Another observation plane was shifted by 6 mm parallel to the center plane to visualize the flow in the native and neo-sinus. The laser sheet was aligned with the observation levels and the camera orthogonal to the observation level as illustrated in Fig. 4. A time interval between the recorded image pairs of dt = 150 µs was chosen in the software (Dynamic Studio 6.7, Dantec Dynamics A/S, Denmark). Images were acquired in a frequency of 14 Hz during the pump cycle. Image data were converted into vector data using an interrogation area of 32 × 32 px and averaging 88 cycles per phase. Disturbances on the inner edge of the aortic membrane were eliminated by applying a coherence filter. The image pairs were recorded by a high-speed camera (CCD-Camera CLM-B241 (Imperx, Inc. Boca Raton, USA) with a Micro Nikkor AF Objective 60 mm f = 2.8 D (Nikon GmbH, Germany)).

### Proof-of-Concept Blood Test

To proof the feasibility of the modified test system with the simulated native leaflets (see “Test Setup”), a comparative blood test was performed. The testing system was developed as a comparative blood study. An original test chamber without native leaflets and neo-sinus and a modified test chamber were tested in parallel with the identical blood from one blood batch. Porcine slaughterhouse blood was used, since its suitability for thrombogenicity tests was shown by Clauser et al. [19] and Schönborn et al. [20] and meets the requirements of DIN EN ISO 5840:1 2021 [20–23].

To allow for a choice between different blood batches, blood from five different donor animals was collected from the jugular vein during the slaughtering process. It was transported in individual bottles on the day of the experiment. The bottles were already prefilled with low molecular weight heparin (LMWH; 2000 IU/mL CLEXANE^®^ multidose 100 mg/mL; Sanofi aventis; Germany) anticoagulating the blood immediately. Before starting the experiment, blood quality from all bottles was evaluated optically and based on various blood parameters. If the blood parameters were outside the defined limits or a clot already formed inside, the bottle was excluded (see Table S1 in the supporting information).

One of the remaining bottles was selected and filled into a 2 L bag (Nutrimix^®^ 2/3, B. Braun, Germany). During transfer, a transfusion filter with a mesh width of 200 μm was used to guarantee that no particles such as micro thrombi or other tissue passed into the chamber. The two test chambers were filled simultaneously. This ensures that the coagulation cascade is not triggered by waiting until the other chamber is filled and connected to the driving unit. After starting the linear piston, aortic pressure and flow were adjusted to physiological conditions and monitored, recorded, and corrected to maintain them throughout the whole experiment.

Adjusting the physiological conditions marked the starting point of the experiment and the first sample (t = 0) was taken. Blood samples were collected every hour until the end of the experiment (t = 4). In addition to the blood samples from the chambers, one sample at the beginning and one at the end (t = 0 and t = 4) were taken from the mixing bag, which was suspended in the climate chamber and served as a control for auto-thrombogenicity. The blood samples were analyzed with the same methods as the blood bottles. To show a decrease because of thrombus formation, the platelet number was measured. Hematocrit as well as Base excess were measured to evaluate that the blood was in physiological condition and not the reason for thrombus formation. To evaluate the state of coagulation, activated clotting time was measured and ROTEM thromboelastometry (ROTEM delta, Tem Innovations GmbH, Germany) was used to analyze the coagulation. For ROTEM, the same analysis methods (Extem, Fibtem and HepNatem) were applied as in Brockhaus et al. [23]. In addition, free plasma hemoglobin was measured to evaluate the hemolytic properties of the valves. After taking the last blood sample at t = 4, the linear piston was stopped, and the test chambers were immediately taken out of the climate chamber. The blood was removed simultaneously and filtered again to detect embolized thrombi.

The prostheses were removed from the test chambers and formed clots on the test devices were fixed using a standardized protocol. The valves were treated with 2.5 wt% glutaraldehyde (Carl Roth GmbH + Co.KG, Germany) to fix the thrombi. Afterwards, the prostheses were rinsed with phosphate buffered saline (PBS) (Lonza Group Ltd., Switzerland) and dried with isopropyl alcohol to analyze the clot mass in dry.

The prostheses were weighted before the experiment and after thrombus fixation to detect the mass of formed thrombi. Size, number, and place of origin of thrombi were analyzed with a light microscope (Keyence VHX-7000, Keyence GmbH, Germany). Blood parameters were analyzed by plotting the differences between the measurement points to the respective starting point of the two test chambers and the blood bag.

## Results

### Hydrodynamics of the PIV and Blood Experiments

**Fig. 5:**
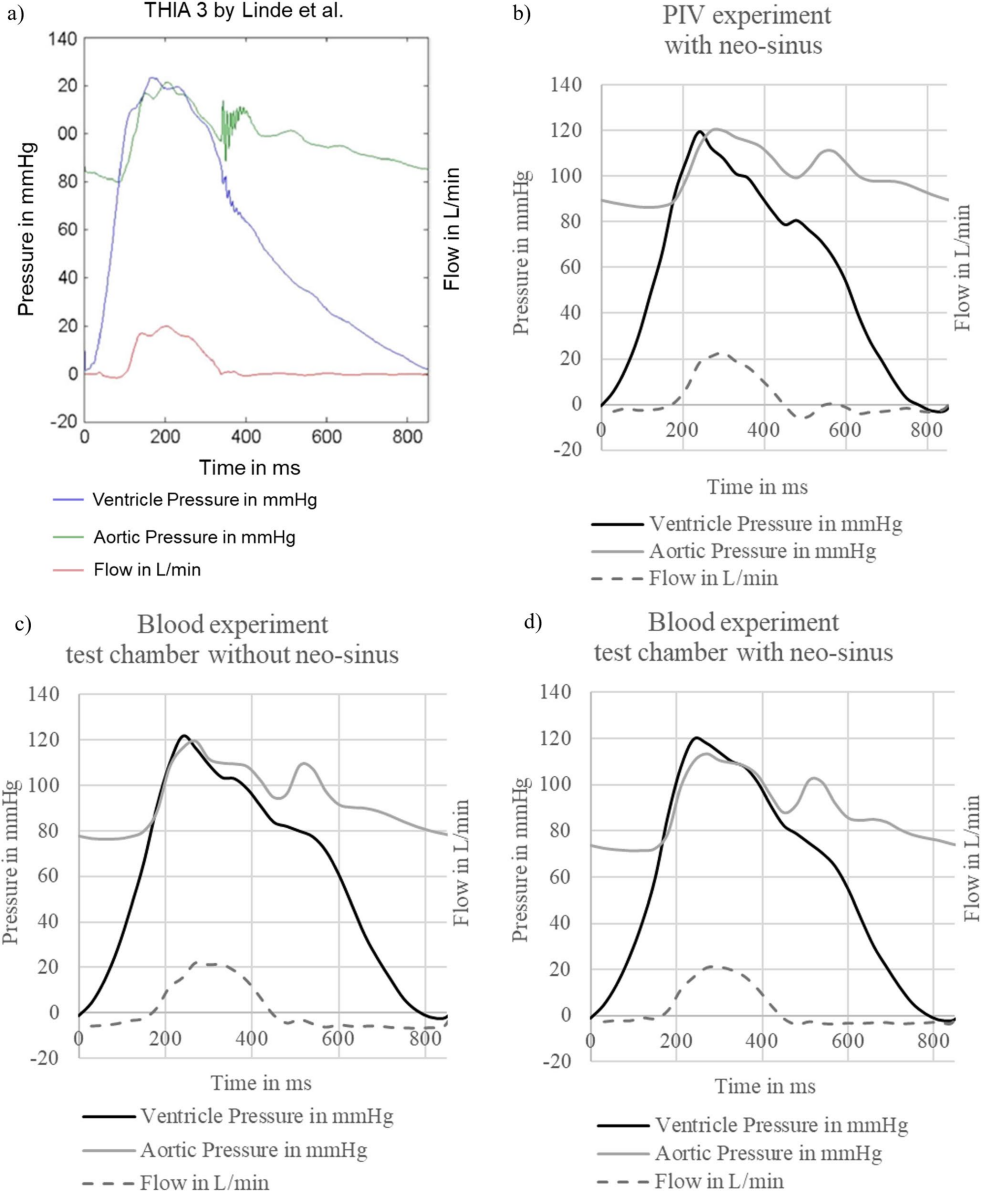
Pressure and flow curves produced **a**) by Linde et al. (Reprint permission license number: 5924141470067), **b**) of the PIV THIA 3, **c**) of the blood experiment with test chamber without neo-sinus and **d**) of the blood experiment with test chamber with neo-sinus

The hemodynamics of the THIA 3 measured by Linde et al. served as reference for this study. Comparing the flow, aortic and ventricular pressure of the reference to those of the experiments in this study, only minor difference was seen between the curve progressions (Fig. 5). As already explained by Linde et al., in all experiments the ventricular pressure slowly decreased to zero until just before the systole began. The comparison of the progression curves of the flow, aortic and ventricular pressure, respectively, between the experiments from this study are illustrated in Fig. S3 in the supporting information.

### PIV Measurements

**Fig. 6:**
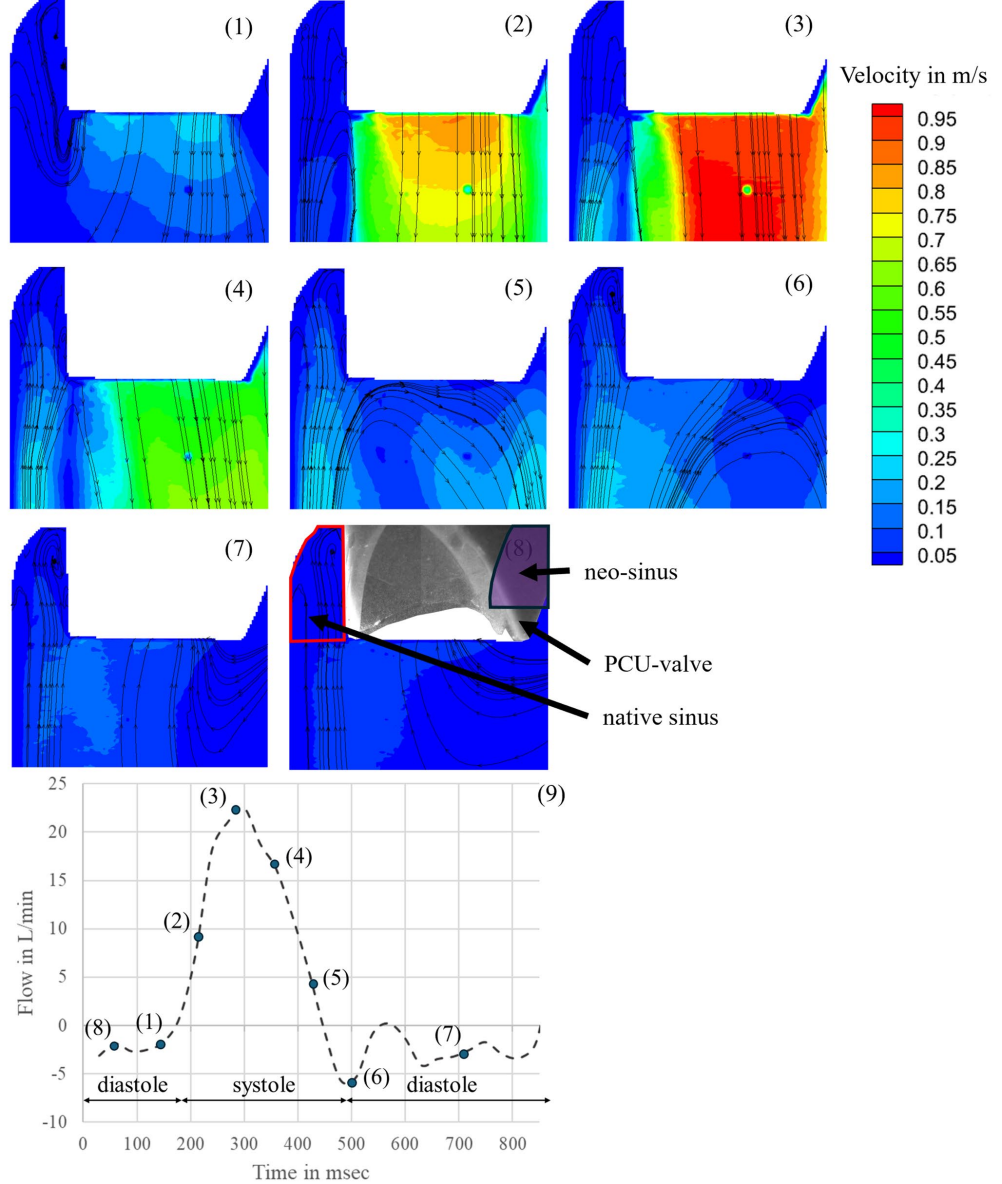
Flow field in the aortic root during one heart cycle in the central plane: (1) end of diastole; (2) beginning of systole; (3) maximum systole; (4) systole; (5) end of systole; (6) beginning of diastole; (7) diastole; (8) diastole; (9) flow through valve showing the selected time points

To validate a physiological flow through the aortic valve including simulated native leaflets and the formed neo-sinus, the velocity field was analyzed at different time points in two observation planes. Figure 6 presents the results for the central plane. The measurement started with the end of diastole (Fig. 6 (1)), when the PCU valve was closed. Due to the opening of the PCU valve, the flow field in the central plane started to build up from the edge of the prosthesis leaflets (Fig. 6 (2)). Then, a clear central jet was developed, and the flow reached its maximum velocity directly behind the prosthesis leaflets with a velocity of 1 m/s (Fig. 6 (3)). At the beginning of the diastole, vortices were formed initiating the valve closure (Fig. 6 (4)). After closure of the PCU valve, no flow was observed (Fig. 6 (5)-(8)).

**Fig. 7:**
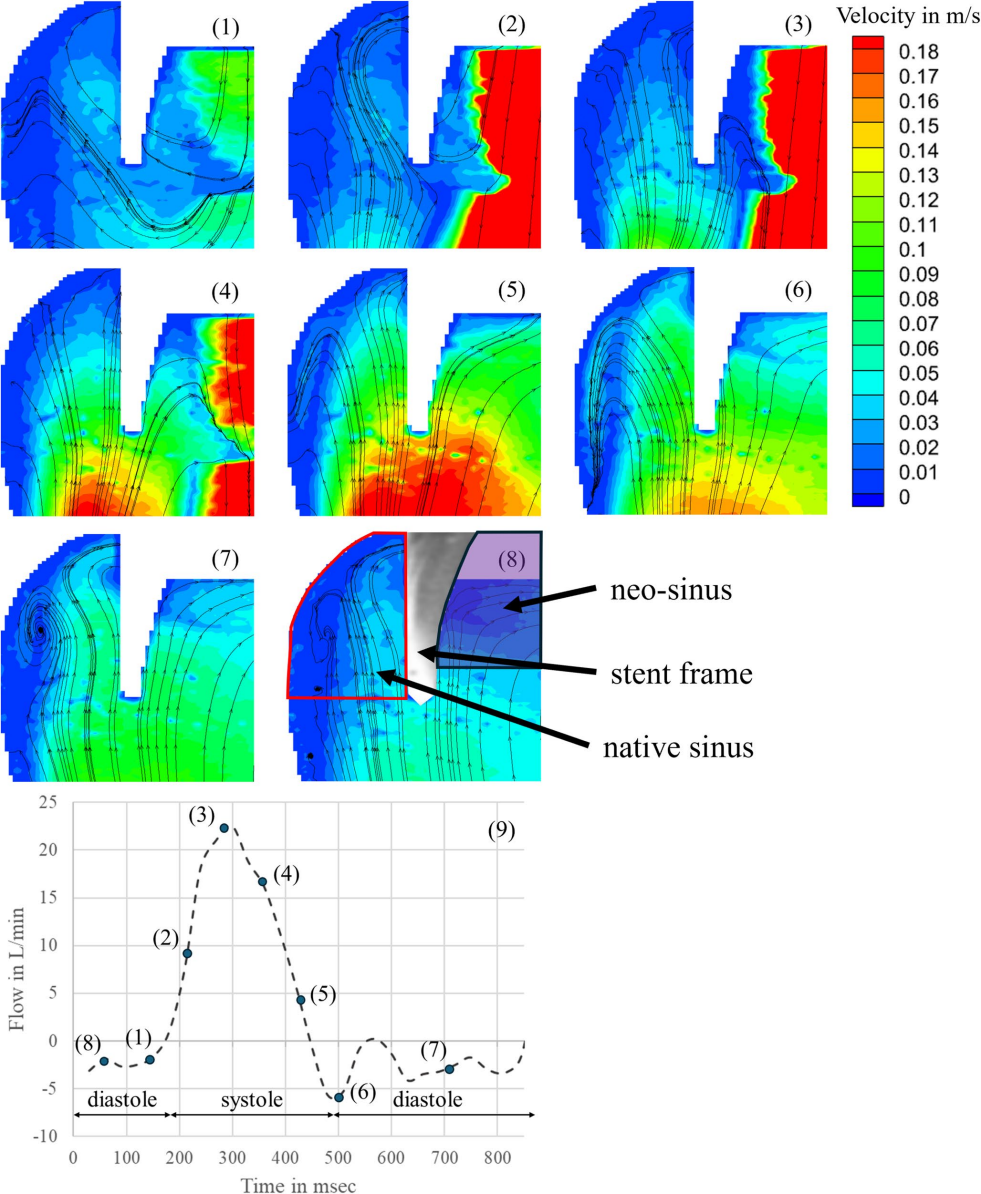
Flow field in the aortic root during one heart cycle at the 6 mm shifted observation plane: (1) end of diastole; (2) beginning of systole; (3) maximum systole; (4) systole; (5) end of systole; (6) beginning of diastole; (7) diastole; (8) diastole; (9) flow through valve showing the selected time points

Figure 7 visualizes the flow field in the native and neo-sinus in the observation plane shifted 6 mm from the central plane. At the end of diastole (Fig. 7 (1)), when the valve is closed, two vortices were established at the bottom of the native sinus. The one next to the native leaflets was clockwise and the other one next to the aortic wall was counterclockwise. The measured velocities were low and ranged from 0 m/s to 0.04 m/s. In the neo-sinus one vortex was visualized, which was clockwise to the native leaflet and the measured velocity was below 0.13 m/s. During valve opening, the two vortices in the bottom of the native sinus and the one in the neo-sinus remained. The velocities in the native sinus decreased below 0.03 m/s. In contrast, the velocities in the neo-sinus increases to a maximum of 0.17 m/s (Fig. 7 (2)). At the peak of systole, a vortex in clockwise direction was observed at the edge of the native leaflet. Instead, the vortex in the neo-sinus was dissolved and the flow was directed downstream with velocities up to 0.17 m/s. The velocities in the native sinus also increased and ranged from 0 m/s to 0.15 m/s, but the flow was directed upstream into the native sinus (Fig. 7 (3)). In Fig. 7 (4), the vortex, that was formed at the edge of the native leaflet, was shifted into the neo-sinus and its velocities as well as the velocities in the native sinus increased, while the main flow through the valve already decreased. At the end of systole, when the valve is already closed, the velocities measured from the edge of the valve in direction of the aorta were up to 0.3 m/s. Two vortices started to build up in the native sinus with a maximum velocity of 0.11 m/s upstream. The vortex next to the aortic wall was counterclockwise and the one next to the native leaflets was clockwise. The vortex in the neo-sinus continued to build up and reached the bottom of the neo-sinus. The flow field was directed upstream with maximum velocities up to 0.11 m/s. (Fig. 7 (5)). At the beginning of the diastole (Fig. 7 (6)), the vortices in the native sinus and the neo-sinus still built up but the velocities started to decrease and ranges between 0 m/s and 0.04 m/s (Fig. 7 (7–8)).

### Blood Experiments

**Fig. 8:**
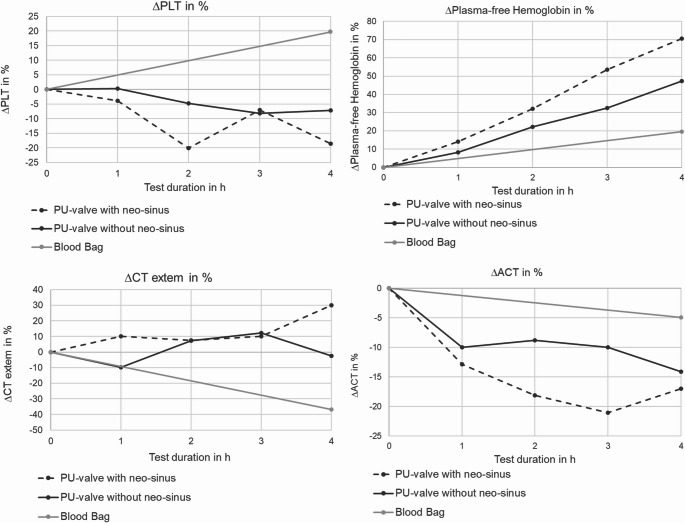
Progression of blood values during blood experiment

The results of blood experiments are shown in Fig. 8 and Fig. S2, respectively. Further results, starting values and the delta between starting and end values are shown in Table S1– S3 in the supporting information.

There were no detectable differences in hematocrit (HCT), cBase (BeOx) and maximum clot firmness (MCF) during the course of the experiment between the test chamber with neo-sinus (neoS), the test chamber without neo-sinus (no-neoS) and the blood bag (BB) (see Figure S2, supplementary information). The platelet numbers (PLT) slightly decreased over time in the two test chambers, with a decrease of 18.53% in the neoS chamber and 7.17% in the no-neoS chamber. In contrast, the static reference presented an increase of nearly 20%. ACT levels constantly decreased in both test chambers and the static reference by a total of 17% for neoS, 14% for no-neoS and 5% for the reference. The levels of plasma-free hemoglobin gradually increased in all cases, namely about 70.5% for neoS, 47.3% for no-neoS and 20% for reference. The clotting time showed a distinct increase at the last sampling point of neoS with a total of 30% and a decrease for the reference of 37%. The no-neoS chamber presented fluctuating values resulting in a slight decrease of 2.44% at the 4 h measurement.

### Evaluation of Thrombus Formation

**Fig. 9:**
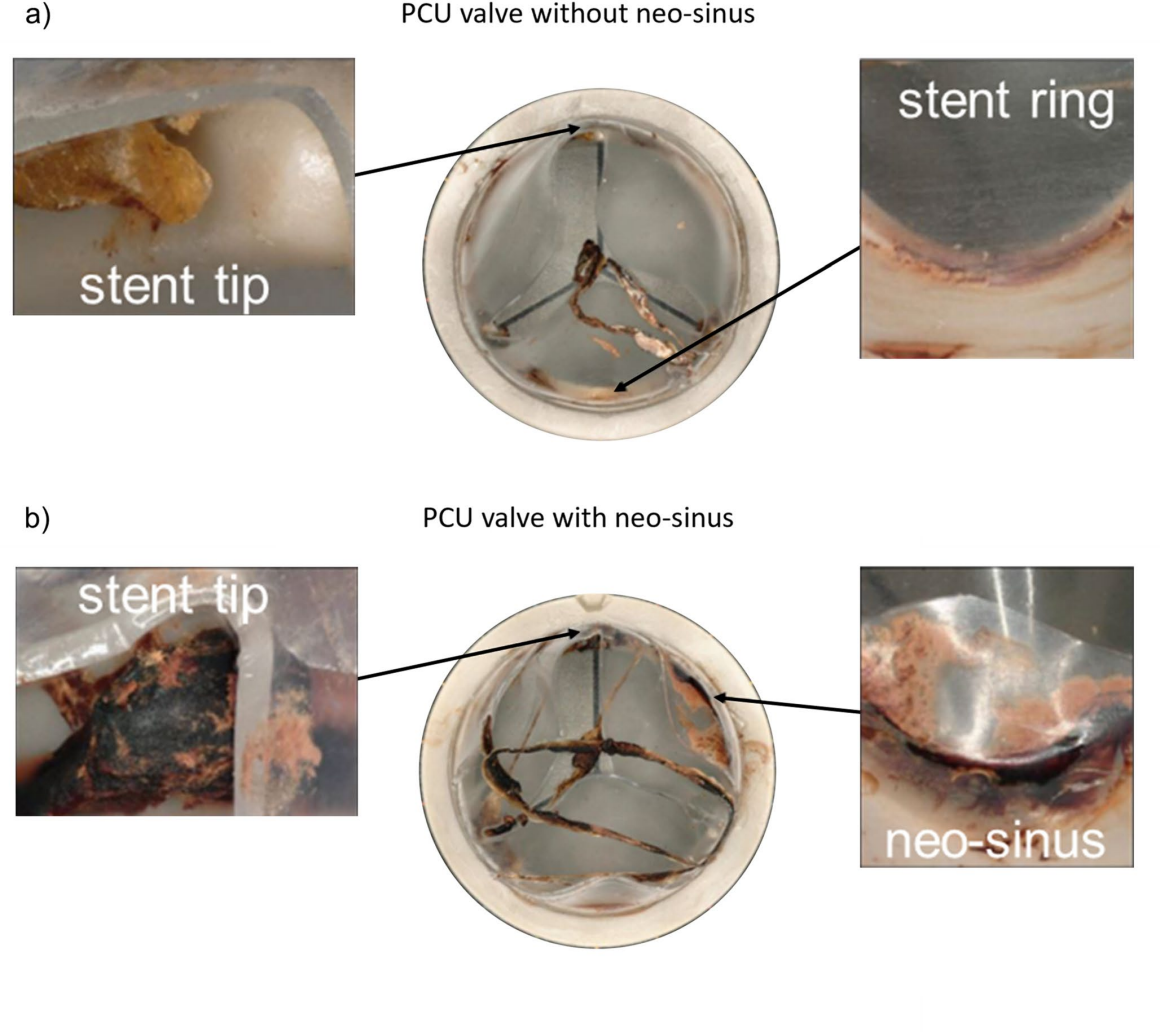
Clot formation (**a**) on the PCU valve without neo-sinus after drying; in detail: stent tip and stent ring and (**b**) on the PCU valve with neo-sinus after drying; in detail: stent tip and neo-sinus

Clots adhered all over both PCU valves but not in the test chambers itself. The major adherence of clots was found between the PCU tube and the stent ring, around the stent tips and at the edge of the upper tube. Smaller thrombi were detected on the leaflets and the stent. The places of origin were the same for the two valves. On the valve with neo-sinus, further clots were formed in the neo-sinus. Additionally, the qualitative number of clots at each position was higher for the valve with neo-sinus resulting in a higher total mass of clots. The determined weight was 0.064 g and 0.0528 g for the valve with and without neo-sinus, respectively, resulting in a 21.21% higher mass of clots on the valve with neo-sinus. The places of origin and the qualitative comparison of both valves are presented in Table 1 and shown in Fig. 9. The evaluation scale reached from “-“ if no clots were found to “+++” if the area was covered with clot material.

**Table 1:** Places of origin and qualitative amount of clots

	Clots on valve with neo-sinus	Clots on valve without neo-sinus
Edge of the upper tube	++	+
Stent tips	+++	++
Stent ring	+	+
Neo-sinus	+++	Not present
Test chamber	-	-
Total mass of clots	0.0640 g	0.0528 g

## Discussion

The topic of thrombus formation in the neo-sinus after TAVR has gained more and more importance in recent years [5–8, 26, 27]. To allow for pre-clinical in-vitro testing of this phenomenon, we adapted a thrombogenicity tester [18] by integrating artificial aortic leaflets into the test chamber to create the neo-sinus.

The PIV experiment visualizes the flow field in the aortic root and the neo-sinus. The validation of a physiological flow field in the aortic root is necessary to attribute possible thrombus formation in the blood experiment to the presence of the neo-sinus. Further, possible risk factors for thrombus formation like stagnation zones in the native and neo-sinus can be analyzed. For both the PIV and the blood experiments, the in-vitro testing system THIA 3, developed by Linde et al., was used and adapted by integrating artificial aortic leaflets into the test chamber to create the neo-sinus. The leaflets were mimicked by a cylindric tube with its upper edge shaped like native commissures made of PCU and pulled over the PCU valve. Furthermore, the results of Linde et al. served as reference for data without neo-sinus.

In the PIV experiment, two observation planes were investigated. One observation plane corresponded to the central plane to validate the physiological flow through the valve (Fig. 6). In this plane, however, the flow in the neo-sinus could not be seen due to the stent frame. Therefore, the other observation plane was shifted by 6 mm parallel to the central plane to visualize the flow in the neo-sinus. The flow field in the native sinus and in the aorta was compared to the results of Linde et al., who investigated the flow in an observation plane shifted by 12 mm to the central plane at the end of systole. From the edge of the valve in direction of the aorta, he measured velocities up to 0.3 m/s like it was also measured here. However, comparing the velocities measured in the native sinus, the maximum velocity in his study was 0.3 m/s, which is higher than the 0.11 m/s measured here while the two observed vortices correspond in their orientation to those seen here. [18] This could be due to the 6 mm difference between the chosen observation planes in his and in our study. Considering the whole cycle, the measured velocities at the bottom of the native sinus do not increase above 0.04 m/s, which is similar to the findings of Madukauwa-David et al. [24]. They measured velocities up to 0.03 m/s in this area also confirmed by further studies finding low velocities and stagnation zones [10, 11, 24, 25].

During systole, two vortices were formed from which the vortex at the bottom of the native sinus was not dissolved during the whole cycle. At the end of systole, one vortex started to build up in the neo-sinus and remained there during diastole. Furthermore, the velocities did not exceed the maximum value of 0.1 m/s, which was also observed by Ducci et al. [25]. They used an Edwards SAPIEN valve (cardiac output 4 L/min and 70 beats/min) in their PIV study and analyzed the flow dynamics in the aortic root in two configurations. One configuration demonstrated the anatomical conditions before TAVR, so that no neo-sinus was present. The other configuration showed the anatomical conditions after TAVR with neo-sinus. By comparing the results of the pre-TAVI configuration, the post-TAVI configuration showed major variations in the aortic root by an extended and prolonged stagnation zone with minimum shear rate in the native sinus [25].

Summarizing the studies mentioned above, all of them showed low velocities, stagnation zones and undissolved vortices in the aortic root as it could be observed in this study. Furthermore, the presence of native leaflets decreases the velocities in the native sinus. A comparison of the velocities measured here to the one by Linde et al. indicates that the presence of native leaflets decreases the velocity in the native sinus. Further, we conclude that the flow dynamics in the aortic root with neo-sinus are realistic in regard to other studies and therefore the test system is suitable for further investigations of the impact of the neo-sinus.

To further proof the test system’s feasibility for comparative thrombogenicity tests with or without a mimicked neo-sinus, a proof-of-concept blood test was conducted and blood parameters were analyzed. The delta in plasma-free hemoglobin showed a distinct difference between both test chambers and the static reference with most increase in hemolysis in the neoS chamber (~ 70%) compared to 50% in the no-neoS chamber and 20% in the static reference. This higher degree of hemolysis might be due to the additional vortices formed in the neo-sinus since recirculation is a known trigger for hemolysis. The decrease in platelet numbers was also more pronounced for the neoS chamber compared to the no-neoS chamber, which is in line with the higher thrombus weight in the neoS heart valve prototype, supporting the impression that more thrombus formation has taken place in the neoS test system. This is supported by the larger increase in the ROTEM clotting time (CT) for the neoS chamber, since an increase in ROTEM hints to a consumption of coagulation factors in the test system and thus more ongoing thrombus formation. In contrast, the MCF does not present differences between neoS and no-neoS chambers. The rise in platelet count and decrease in CT for the static reference appears to be a measurement error since platelet numbers cannot increase over time during an in-vitro test. Both values could be attributed to not perfect mixing of the static reference before sample drawing, which needs to be improved in the future. The constant decrease of ACT in all three circuits is reasonable since anticoagulation is consumed over time and coagulation processed, as indicated by the other blood parameters. Maybe, the 4 h value of the neoS chamber is due to the measurement inaccuracy of the ACT device and is even lower than measured here. This would be in line with the other blood parameters, indicating a higher degree of coagulation activation in the neoS chamber.

Since the study is a proof-of-concept study, no repetitions of the blood experiments were performed and therefore a proper statistical analysis of the blood value progression is not possible. Further, other blood parameters, especially for early coagulation initiation such as platelet activation markers, FXII or FXa could be evaluated in future studies, which might be more sensitive to slight differences in thrombotic activity and increase the significance of the results.

However, differences in the number of clots between the valve with neo-sinus and without neo-sinus could be detected, namely more clots on the valve with neo-sinus. Additional clots also formed inside the neo-sinus itself. This is also reflected by the total mass of clots, which is 21.21% higher for the neoS valve. A benchmark with in-vivo study by Hansson et al. and Makkar et al., who examined a large cohort with more than 460 and 130 patients, respectively, showed similar places of thrombi origin as in our test [26, 27]. Since the evaluation of the adherent thrombi was carried out manually and qualitatively, in the future other imaging techniques such as µCT could be used to gain more insights on the exact spots of thrombus formation on the valves.

Although our study is limited by relying on one single blood experiment, the results still indicate a higher thrombogenic potential in the test system with neo-sinus and proves overall feasibility of using the adapted thrombogenicity tester with mimicked native aortic leaflets and created neo-sinus for thrombogenicity evaluation of TAVR valves. In a future benchmark study using clinically approved heart valves, the number of test repetitions can be increased and results can be compared to existing in -vivo data. Furthermore, additional coagulation parameters can be included in the analyses to depict coagulation kinetics over the experiment duration. In the future, the test system will strongly contribute to pre-clinical assessment of thrombogenicity of transcatheter heart valve prostheses and will therefore help to overcome subclinical thrombus formation after TAVR.

## Conclusion

The aim of this study was to mimic the natural environment after TAVR in vitro in order to demonstrate that the presence of a neo-sinus increases the thrombogenic risk of transcatheter heart valves. In the proof-of-concept blood test, clots formed in the same regions as reported in in-vivo studies in literature. The PIV experiment revealed a physiological flow field in the native sinus as well as in the neo-sinus with low velocities and stagnation in the neo-sinus, thereby proving our test system a replication of the physiological environment of transcatheter heart valves. An in-vitro blood test comparing the test system without neo-sinus to the version with neo-sinus showed about 20 wt% more thrombotic adhesion on the valve from the neo-sinus tester, showing the impact of the neo-sinus for the first time in an in-vitro blood test.

Once established as a validated method, this approach can be used in the early stages of THV development to optimize designs with reduced thrombogenic risk, prior to in vivo testing—thereby improving patient safety.

## Electronic Supplementary Material

Below is the link to the electronic supplementary material.

Supplementary Material 1

## Data Availability

Additional data is available as supporting information.
