# Supplementary material for: Role of Neo-Sinus on Thrombogenicity of Aortic Valve Prostheses: Experimental Proof-of-Concept Study
Source: Cardiovasc Eng Technol. 2025 Jul 14;16(5):537–50. doi: 10.1007/s13239-025-00792-z (PMC12528357; doi:10.1007/s13239-025-00792-z)
Supplement: Supplementary file 1 — Supplementary Material 1 [file 13239_2025_792_MOESM1_ESM.pdf]

Supporting Information for Cardiovascular Engineering and Technology:

# Role of neo-sinus on thrombogenicity of transcatheter heart valve prostheses: experimental proof-of-concept study

Saskia Thoenissen[1], Ilona Mager[1], Claudio A. Luisi[1], Markus Mous[1], Thomas Schmitz-Rode[2], Ulrich Steinseifer[1], Johanna C. Clauser[1]

[1] Department of Cardiovascular Engineering, Institute of Applied Medical Engineering, Medical Faculty, RWTH Aachen University Hospital, Aachen, Germany

[2]Institute of Applied Medical Engineering, Medical Faculty, RWTH Aachen University, Aachen, Germany

Corresponding author

Johanna C. Clauser
Address correspondence to J. Clauser, Department of Cardiovascular Engineering, Institute of Applied Medical Engineering, Medical Faculty, RWTH Aachen University, Forckenbeckstr. 55, 52074 Aachen, Germany.
Electronic mail: clauser@ame.rwth-aachen.de
Orcid Number: https://orcid.org/0000-0002-9050-2391

*Table S1: Blood parameters for the selection of blood donations*

| **Parameter** | **Target values** | **Allowed deviation** | **Initial values of chosen blood donation** |
|---|---|---|---|
| Platelet number (PLT) | 280 x $10^3$ /µL | ± 50 x $10^3$ /µL | 305 x $10^3$ /µL |
| Hematocrit (HCT) | 40 % | ± 4 % | 37.8 % |
| HepNatem Clotting Time (CT) | < 1300 s | - | 635 s |
| pH | 7.400 | ± 0.100 | 7.371 |
| cBase ($Be_{ox}$) | 0 mmol/L | ± 5 mmol/L | 0.6 mmol/L |

*Table S2: Blood parameters at start of the experiment*

| | **Test chamber with neo-sinus (TC1)** | **Test chamber without neo-sinus (TC2)** | **Blood bag** |
|---|---|---|---|
| PLT/($10^3$/µL) | 313 | 293 | 320 |
| HCT/% | 37.1 | 37 | 37 |
| CT extem/s | 40 | 41 | 57 |
| MCF extem/s | 67 | 67 | 66 |
| cBase (BeOx)/(mmol/L) | -0.9 | -0.6 | 0.1 |

| | | | |
|---|---|---|---|
| ACT/s | 171 | 170 | 163 |
| Plasma-free hemoglobin/(mg/dL) | 23.965 | 24.480 | 25.365 |

*Table S3: Difference of blood parameters from experiment start to end*

| | **Test chamber with neo-sinus (TC1)** | | **Test chamber without neo-sinus (TC2)** | | **Blood bag** | |
|---|---|---|---|---|---|---|
| ΔPLT | -58 x 10³/µL | -18.53 % | -21 x 10³/µL | -7.17 % | 63 x 10³/µL | 19.69 % |
| ΔHCT | 0.5 % | 1.35 % | 0.8 % | 2.16 % | -10.5 % | -28.38 % |
| ΔCT extem | 12 s | 30.00 % | -1 s | -2.44 % | -21 s | -36.84 % |
| ΔMCF extem | -2 s | -2.99 % | -1 s | -1.49 % | 6 s | 9.09 % |
| ΔcBase (BeOx) | -1.6 mmol/L | 177.78 % | -1.7 mmol/L | 283.33 % | -1.9 mmol/L | -1900.00 % |
| ΔACT | -29 s | -16.96 % | -24 s | -14.12 % | -8 s | -4.91 % |
| ΔPlasma-free hemoglobin | 16.90 mg/dL | 70.52 % | 11.58 mg/dL | 47.30 % | 4.975 mg/dL | 19.61 % |

*Table S4: Comparison of the different test setups of the THIA 3*

| | **Blood-THIA 3** | | **PIV-THIA 3** |
|---|---|---|---|
| | **Test chamber with neo-sinus** | **Test chamber without neo-sinus** | |
| used valve | in-house designed PCU-Valve | in-house designed PCU-Valve | in-house designed PCU-Valve |
| diameter of the valve | 25 mm | 25 mm | 25 mm |
| aortic root | | | |
| presence/absence of neo-sinus | presence of neo-sinus | absence of neo-sinus | presence of neo-sinus |
| material for native leaflets | PCU | NA | PCU |
| replication of the native leaflets | tube with its upper edge shaped like native commissures | NA | tube with its upper edge shaped like native commissures |
| test fluid | blood | blood | water-glycerol |
| aortic inlay | adopted from Linde et al. | adopted from Linde et al. | adopted from Linde et al. |

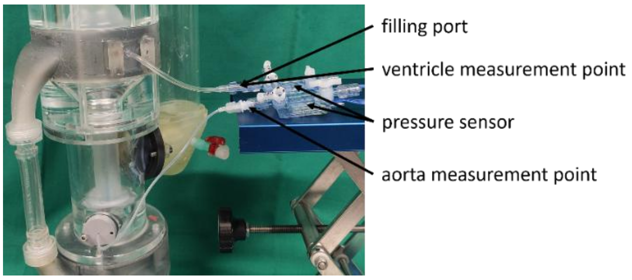


*Figure S1: Filling port and pressure measurement points of the modified THIA3*

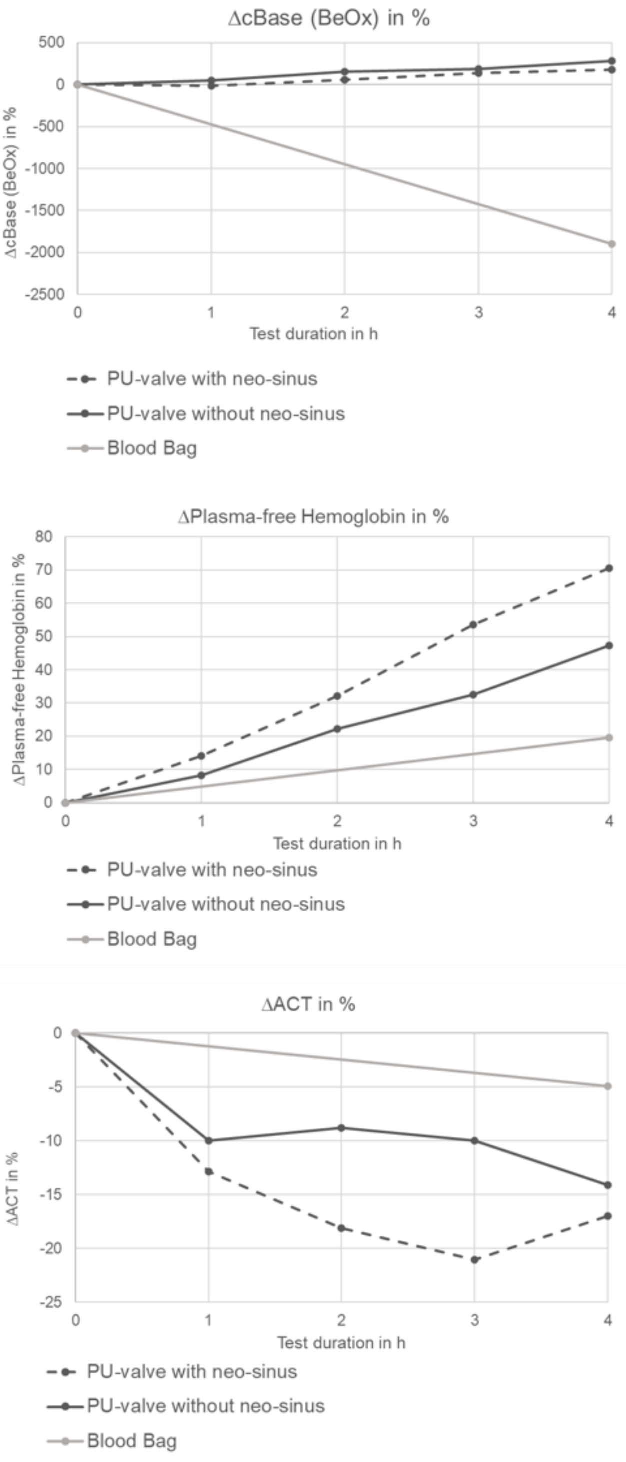


*Figure S2: Blood values during the 4 h blood experiment*

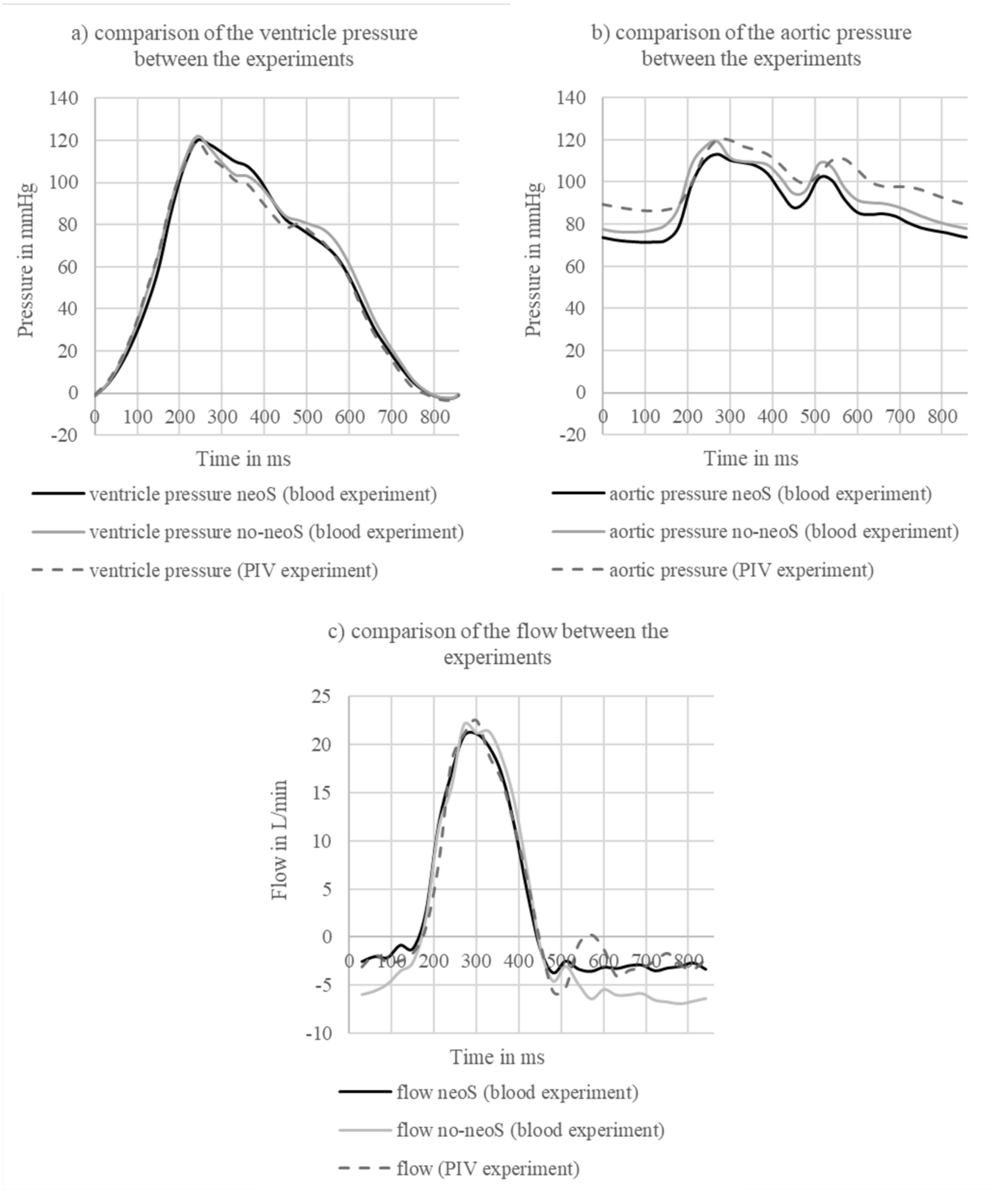


*Figure S3: Comparison of the a) ventricle pressure; b) aortic pressure and c) flow curves of PIV and blood experiments*
